# Morphology and multi-locus phylogeny reveal three new *Cortinarius* (Cortinariaceae, Agaricales) species from northwestern Yunnan, China

**DOI:** 10.3897/mycokeys.134.197559

**Published:** 2026-06-24

**Authors:** Yi-Wen Yang, Qi Zhao, Ying Zhang

**Affiliations:** 1 Key Laboratory of Forest Disaster Warning and Control in Yunnan, College of Forestry, Southwest Forestry University, Kunming, Yunnan 650224, China State Key Laboratory of Phytochemistry and Natural Medicines, Yunnan Key Laboratory for Fungal Diversity and Green Development, Kunming Institute of Botany, Chinese Academy of Sciences Kunming China; 2 State Key Laboratory of Phytochemistry and Natural Medicines, Yunnan Key Laboratory for Fungal Diversity and Green Development, Kunming Institute of Botany, Chinese Academy of Sciences, Kunming, Yunnan 650201, China College of Forestry, Southwest Forestry University, Kunming Kunming China; 3 College of Biological Science and Food Engineering, Southwest Forestry University, Kunming, Yunnan 650224, China College of Biological Science and Food Engineering, Southwest Forestry University Kunming China

**Keywords:** Morphology, novel species, phylogeny, sect. *Anomali*, subg. *Leprocybe*

## Abstract

Yunnan’s complex topography and pronounced geographic heterogeneity, together with marked climatic variability, luxuriant vegetation, and the seasonal influx of warm, moisture-laden air masses from the Indian and Pacific Oceans, have created abundant refugial habitats throughout the Quaternary glaciations. These combined abiotic and biotic factors have made the region highly favorable for the survival, diversification, growth, and reproduction of higher fungi. This study reports three new species: *Cortinarius acuticonus*, *C. tricholomopsoides*, and *C. wudihuensis* from the subalpine and alpine forests of northwestern Yunnan, China, identified through morphological investigations and multi-locus phylogenetic analyses. *Cortinarius acuticonus* and *C. tricholomopsoides* are placed in the subgenus *Leprocybe*. *Cortinarius acuticonus* is distinguished by an acutely conical pileus with dense black, *Strobilomyces*-like scales and a distinct membranous annulus. In contrast, *Cortinarius tricholomopsoides* exhibits large, pale yellow basidiomata and forms associations with *Abies* species. *Cortinarius wudihuensis*, assigned to section *Anomali*, is characterized by small basidiomata with violaceous to brownish tones, an association with *Quercus aquifolioides*, and larger, broadly ellipsoid basidiospores (7–9 × 5.5–7 μm). These findings underscore the substantially underestimated diversity of *Cortinarius* in the high-altitude regions of Yunnan and offer important insights into the ecological specialization and evolutionary divergence of Asian *Cortinarius* lineages.

## Introduction

Northwestern Yunnan stands as one of the world’s most remarkable biogeographical transition zones, where the eastern escarpment of the Qinghai–Tibet Plateau descends through successive vegetation belts into subtropical valleys incised by the Jinsha, Lancang, and Nujiang river systems (Lu et al. 2026). This tri-river landscape, designated by the United Nations Educational, Scientific and Cultural Organization (UNESCO) as a World Heritage Site, forms a sharply compressed altitudinal gradient exceeding 5,000 m within horizontal distances of only several tens of kilometers, thereby generating an exceptional diversity of microhabitats (Liu et al. 2024).

*Cortinarius* (Pers.) Gray is the most species-rich genus within Agaricales (Tedersoo and Nara 2010; Soop et al. 2019), and it plays a critical role in forest ecosystems as an ectomycorrhizal symbiont with a global distribution (Bödeker et al. 2014; Martin et al. 2016). Despite its ecological prominence, the taxonomy of the genus has historically been impeded by its immense diversity and morphological complexity (Soop et al. 2019). Traditionally, infrageneric classification relied heavily on macromorphology and chemotaxonomy. Liimatainen et al. (2022a) proposed a refined classification system for the genus *Cortinarius* by integrating genomic analysis with multi-gene phylogenetics. This updated system delineates 11 subgenera: *Cortinarius* subg. *Cortinarius* (Pers.) Gray, *Camphorati* Liimat., Niskanen & Ammirati, *Dermocybe* (Fr.) Trog, *Illumini* Liimat., Niskanen & Kytöv., *Infracti* Niskanen & Liimat., *Iodolentes* Niskanen & Liimat., *Leprocybe* M.M. Moser, *Myxacium* (Fr.) Trog, *Orellani* (M.M. Moser) Gasparini, *Paramyxacium* M.M. Moser & E. Horak, and *Telamonia* (Fr.) Trog (Moser 1969; Niskanen and Kytövuori 2008; Liimatainen 2014; Liimatainen et al. 2015, 2017, 2020, 2022a; Niskanen et al. 2016).

In recent decades, the integration of molecular phylogenetics has changed the understanding of *Cortinarius* systematics (Lindahl et al. 2013; Liimatainen 2014; Liimatainen et al. 2015, 2017, 2020, 2022a). The application of DNA barcoding, particularly using the nuclear ribosomal internal transcribed spacer (ITS) region, has revealed extensive cryptic diversity across various infrageneric groups (Lindahl et al. 2013). Recent studies in different geographical regions have demonstrated that many widely distributed names encompass multiple distinct phylogenetic species, many of which remain undescribed (Skrede et al. 2011; Harrower et al. 2015). This is particularly evident in regions such as East Asia, where intensive sampling and molecular analyses continue to uncover a wealth of novel taxa (Liimatainen et al. 2022a; Xie et al. 2025).

During ongoing investigations of fungal diversity in China (Zhou et al. 2022; Zeng et al. 2023; Su et al. 2025; Wang et al. 2025; Li et al. 2025, 2026; Lu et al. 2026; Zhou et al. 2026), two distinct *Cortinarius* populations belonging to subg. *Leprocybe* and sect. *Anomali* were collected. Morphological and molecular phylogenetic analyses substantiate that these collections constitute three previously undescribed species. Herein, these three new species of *Cortinarius* are formally described, with illustrations, taxonomic comparisons, and a discussion of their phylogenetic positions within the genus.

## Materials and methods

### Sample collection

Specimens were collected from Laojun Mountain Nature Reserve in Lijiang City and Jinguangsi Nature Reserve in Yongping County, Dali, Yunnan Province. Fresh basidiomata were photographed *in situ* prior to collection; collection details were noted (Rathnayaka et al. 2025); and subsequently, they were transported to the laboratory in plastic collection boxes. All specimens were examined according to the methods described by Zhao et al. (2015) and Yang et al. (2025), with specific procedures detailed below. Macroscopic features of the fresh basidiomata were recorded and described based on *in situ* photographs, with color codes following Kornerup and Wanscher (1981). The basidiomata were dried in an electric dryer at 40 °C until completely dehydrated for preservation. The specimens were deposited in the Herbarium of Cryptogams of Kunming Institute of Botany, Chinese Academy of Sciences (KUN-HKAS).

### Morphological observation

Microscopic features were examined and photographed using a stereomicroscope (SteREO Discovery.V12, Carl Zeiss Microscopy GmbH, Germany) and a compound microscope (Nikon ECLIPSE 80i, Nikon, Japan) equipped with a Nikon DS-Ri2 digital camera (Nikon, Japan). For microscopic observation, thin sections were prepared by hand using a razor blade and placed on sterile filter paper moistened with water to maintain hydration. Sections were then mounted in 10% KOH and stained with Congo Red when necessary. Microscopic characteristics were recorded, with at least 30 basidiospores and 20 basidia measured for each mature specimen. To observe basidiospore ornamentation, small hymenophoral fragments were taken from dried specimens, mounted on aluminum stubs with double-sided adhesive tape, coated with gold-palladium, and examined under a ZEISS Sigma 300 scanning electron microscope (SEM) at the Kunming Institute of Botany, Chinese Academy of Sciences (Jia et al. 2025). Microstructures were measured using Image Frame Work v.0.9.7 software. Measurements are presented as (a–) b–c (–d), where a denotes the minimum value, d the maximum value, and b–c the 90% confidence interval. The number of basidiospores (*n*), basidiomata (*m*), and specimens (*p*) measured is denoted as [*n/m/p*]. Q represents the length-to-width ratio of individual basidiospores, and **Q** (referring to the mean value here) represents the average of these ratios ± standard deviation. Illustrations were created using Adobe Photoshop 2022 (Adobe Systems, USA).

### DNA extraction, PCR amplification, and sequencing

DNA was extracted directly from *Cortinarius* fruiting bodies using a *Trelief*™ Fungal Genomic DNA Extraction Kit (Tsingke Biotechnology Co., Ltd., Beijing, China). The nuclear ribosomal internal transcribed spacer (ITS) region and the nuclear ribosomal large subunit (nrLSU) were amplified using the universal primer pairs ITS1/ITS4 (White et al. 1990) and LR0R/LR5 (Vilgalys and Hester 1990), respectively. The polymerase chain reaction (PCR) protocol consisted of an initial denaturation at 95 °C for 5 min, followed by 35 cycles of denaturation at 95 °C for 30 s, annealing at 53 °C for 20 s, and extension at 72 °C for 30 s, with a final extension step at 72 °C for 10 min. PCR products were sequenced by Tsingke Biotechnology Co., Ltd. (Beijing, China).

### Sequence alignment and phylogenetic analyses

Newly generated sequences were verified and assembled using DNAMAN v.9.0.1.116 (Lynnon BioSoft). To construct phylogenetic trees, these sequences were combined with those of closely related species retrieved from GenBank (Tables 1, 2). Multiple sequence alignment was performed using MAFFT v.7 (Katoh and Standley 2013; Katoh et al. 2019) and trimmed with TrimAl v.1.3 (Capella-Gutiérrez et al. 2009), with a gap threshold of 0.5 for both the ITS and nrLSU regions. The trimmed sequences were concatenated into a combined dataset using Sequence Matrix v.1.8 (Vaidya et al. 2011).

**Table 1.** Names, voucher numbers, countries, references, and corresponding GenBank accession numbers of the taxa used in this study. Names shaded in blue indicate newly described species in this study. All type specimens are highlighted with “**T**.” “—” indicates missing data.

Species	voucher	Locations	GenBank Accession No.		Reference
			ITS	nrLSU	
*Cortinarius acuticonus* **T**	KUN-HKAS 151741	China	PX765862	PX765871	This study
*C. acuticonus*	KUN-HKAS 151743	China	PX765863	PX765872	This study
*C. acuticonus*	KUN-HKAS 151742	China	PX765864	PX765873	This study
*C. apius* **T**	WTU:3460	Canada	MW009182	—	Ammirati et al. (2021)
*C. atkinsiae* **T**	TENN:F-071879	USA	NR_173275	—	Ammirati et al. (2021)
*C. atrosquamosus*	H:T. Niskanen 12-278	USA	MW009183	—	Ammirati et al. (2021)
*C. aureopigmentatus*	JFA 11940	Costa Rica	EF420140	—	Ammirati et al. (2007)
*C. clandestinus*	WTU:J.F. Ammirati 9286	USA	MW009213	—	Ammirati et al. (2021)
*C. cotoneus* **T**	S:H. Lindstrom CFP1032	Sweden	MW009216	—	Ammirati et al. (2021)
*C. cf. cotoneus*	HMAS260331	China	KX513578	—	Bidaud et al. (2021)
*C. cf. cotoneus*	ZWL560	China	KX444284	—	Bidaud et al. (2021)
*C. flavifolius* **T**	TENN:068695	USA	MW009217	—	Ammirati et al. (2021)
*C. fuscoflavidus* **T**	WTU:J.F. Ammirati 11644	USA	MW009221	—	Ammirati et al. (2021)
*C. fuscotomentosus* **T**	DBB00566	USA	MT853246	—	Ammirati et al. (2021)
*C. griseoaurantinus* **T**	HMAS 353381	China	PQ796783	PV174519	Dang et al. (2025)
*C. griseoaurantinus*	HMAS 353382	China	PQ796784	PV174520	Dang et al. (2025)
*C. hengduanensis* **T**	HMAS:250455	China	KX513581	—	Hong et al. (2024)
*C. hengduanensis*	KUN-HKAS 151746	China	PX765866	PX765877	This study
*C. hengduanensis*	KUN-HKAS 151747	China	PX765865	PX765876	This study
*C. hughesiae* **T**	TENN:J.F. Ammirati 13086	USA	MW009224	—	Ammirati et al. (2021)
*C. jimenezianus* **T**	JA:CUSSTA9402	Spain	NR_186956	—	Bidaud et al. (2021)
*C. leproleptopus* **T**	PC:R. Henry 84.109	France	MW009226	—	Ammirati et al. (2021)
*C. loringii* **T**	WTU:SCL6030	USA	MW009227	—	Ammirati et al. (2021)
*C. lutescens* **T**	NYS:f1781	USA	MW009228	—	Ammirati et al. (2021)
*C. melanotus* **T**	S:H. Lindstrom CFP1101	France	NR_172988	—	Ammirati et al. (2021)
*C. nigrosquamosus*	HMJAU 58929	China	PZ274249	—	Xie (2022)
*C. nigrosquamosus*	HMJAU 49111	China	PZ274250	—	Xie (2022)
*C. olivaceosquamosus* **T**	H:T. Niskanen 10-105	Canada	MW009231	—	Ammirati et al. (2021)
*C. parkeri* **T**	ADP000-502-1 (WTU)	USA	JN976987	—	Ammirati et al. (2012)
*C. pescolanensis* **T**	MCVE:29054	Italy	NR_153070	—	Picillo and Marchionni (2016)
*C. phrygianus* **T**	S:H. Lindstrom CFP774	Sweden	MW009234	—	Ammirati et al. (2021)
*C. selinolens* **T**	MPU:1116858	France	NR_184930	—	Bidaud et al. (2021)
*C. squamivenetoideus* **T**	H:T. Niskanen 11-005	USA	MW009239	—	Ammirati et al. (2021)
*C. squamivenetus* **T**	H:6001874	Finland	NR_131864	—	Liimatainen (2014)
*C. subcotoneus* **T**	PML 2143	France	MW010122	—	Bidaud et al. (2021)
*C. subleproleptopus* **T**	H:T. Niskanen 12-344	USA	MW009241	—	Ammirati et al. (2021)
*C. tricholomopsoides* **T**	KUN-HKAS 151739	China	PX765860	PX765874	This study
*C. tricholomopsoides*	KUN-HKAS 151740	China	PX765861	PX765875	This study
*C. veneto-occidentalis* **T**	H:T. Niskanen 11-051	USA	MW009243	—	Ammirati et al. (2021)
*C. venetus* **T**	S:H. Lindstrom CFP112	Sweden	MW009250	—	Ammirati et al. (2021)
*C. cf. venetus*	2M06	Japan	LC373240	—	Bidaud et al. (2021)
*C. cf. venetus*	HMAS:274611	China	KX513584	—	Bidaud et al. (2021)
*C. cf. venetus*	HMAS:268596	China	KX513586	—	Bidaud et al. (2021)
*C. veronicae* **T**	PDD 68468	New Zealand	NR_157917	—	Bidaud et al. (2021)
*C. veronicoides*	MEL2120747	Australia	GQ890324	—	Danks et al. (2010)
*C. viridans* **T**	MPU:1116859	Cyprus	NR_184931	—	Bidaud et al. (2021)
*C. yadingensis* **T**	HMAS:254819	China	OR538892	—	Hong et al. (2024)

**Table 2.** Names, voucher numbers, countries, references, and corresponding GenBank accession numbers of the taxa used in this study. Names shaded in blue indicate newly described species in this study. All type specimens are highlighted with “**T**.” “—” indicates missing data.

Taxa	voucher	Locations	GenBank Accession No.		Sequence origin
			ITS	nrLSU	
*Cortinarius adrianae*	UCH CO5272	Panama	OP265184	—	Liimatainen et al. (2022b)
*C. albidipes* **T**	NYS-F-000129	USA	MZ580485	—	Dima et al. (2021)
*C. albidoavellaneus* **T**	MICH10313	USA	MZ580483	—	Dima et al. (2021)
*C. albocyaneoides* **T**	HMJAU 48664	China	ON254420	OR105105	Xie et al. (2025)
*C. albocyaneus*	HMJAU 44500	China	ON254434	OR105121	Xie et al. (2025)
*C. albocyaneus* **T**	CFP1177	Sweden	KX302206	—	Dima et al. (2016)
*C. albomalus* **T**	H7000816	Canada	MZ568645	—	Liimatainen and Niskanen (2021)
*C. anocorium* **T**	H7068022	USA	MZ568646	—	Liimatainen and Niskanen (2021)
*C. anomalobrunneus* **T**	UCH AC653	Panama	OP265185	—	Liimatainen et al. (2022b)
*C. anomalodelicatus* **T**	JFA8146	USA	MZ580480	—	Dima et al. (2021)
*C. anomalomontanus* **T**	JFA9919	USA	MZ580478	—	Dima et al. (2021)
*C. anomalopacifcus* **T**	DBB11745	USA	MZ663774	—	Dima et al. (2021)
*C. anomalovelatus* **T**	JFA13109	USA	FJ717605	FJ717605	Harrower et al. (2011)
*C. anomalus* **T**	CFP1154	Sweden	KX302224	—	Dima et al. (2016)
*C. azureovelatus*	HMJAU 48656	China	ON254406	OR105092	Xie et al. (2025)
*C. azureovelatus*	LE315531	Russia	MN308204	—	Brandrud et al. (2019)
*C. barlowensis* **T**	JFA13140	USA	FJ717554	FJ717554	Harrower et al. (2011)
*C. brevissimus* **T**	NYS-F-000541	USA	MZ580467	—	Dima et al. (2021)
*C. bolaris* **T**	CFP1008	Sweden	KX302233	—	Dima et al. (2016)
*C. bolaris*	TUB 0118524	Germany	AY669596	AY669596	Garnica et al. (2005)
*C. caeruleoanomalus* **T**	JFA13084	USA	MZ663780	—	Dima et al. (2021)
*C. caesiifolius* **T**	MICH10326	USA	MZ580462	—	Dima et al. (2021)
*C. campanianomalus*	HMJAU 48693	China	ON254474	OR105160	Xie et al. (2025)
*C. campanianomalus* **T**	HMJAU 48748	China	ON254479	OR105165	Xie et al. (2025)
*C. caninus*	HMJAU 44258	China	ON254441	OR105129	Xie et al. (2025)
*C. caninus* **T**	CFP627	Sweden	KX302250	—	Dima et al. (2016)
*C. cinnamomeolilacinus* **T**	TCWH 007	China	OQ913384	—	Xie et al. (2025)
*C. cinnamomeolilacinus*	Li 161015-10	China	OQ913385	—	Xie et al. (2025)
*C. clackamasensis* **T**	JFA11616	USA	MZ580452	—	Dima et al. (2021)
*C. clintonianus* **T**	NYS-F-000786	USA	MZ580450	—	Dima et al. (2021)
*C. deceptivus* **T**	MICH10343	USA	MZ663788	—	Dima et al. (2021)
*C. durifoliorum* **T**	PDD101829	New Zealand	KJ635210	MW263597	Soop et al. (2018)
*C. epsomiensis*	HMJAU 44242	China	ON254422	OR105107	Xie et al. (2025)
*C. epsomiensis* **T**	K(M)74963	UK	MK010952	—	Liimatainen and Ainsworth (2018)
*C. harvardensis* **T**	NL-5415	USA	MZ663789	—	Dima et al. (2021)
*C. jonimitchelliae* **T**	HL03-339	Sweden	KX302253	—	Dima et al. (2016)
*C. kranabetteri*	HMJAU 48644	China	ON254398	OR105086	Xie et al. (2025)
*C. kranabetteri* **T**	TN11-287	USA	MZ580449	—	Dima et al. (2021)
*C. latiodistributus* **T**	DB6139	Sweden	MZ663791	—	Dima et al. (2021)
*C. lepidopus*	HMAS 271997	China	ON254483	OR105169	Xie et al. (2025)
*C. lepidopus*	HMAS 272035	China	ON254484	OR105170	Xie et al. (2025)
*C. lividomalvaceus* **T**	JMT-15102001	France	KY315416	—	Zhang et al. (2023)
*C. luteoperonatus*	JMB2011112610	France	KY315415	—	Zhang et al. (2023)
*C. microalbocyaneus*	HMJAU 48649	China	ON254461	OR105147	Xie et al. (2025)
*C. microalbocyaneus* **T**	HMJAU 48706	China	ON254462	OR105148	Xie et al. (2025)
*C. modestus* **T**	NYS-F-001966	USA	MZ580446	—	Dima et al. (2021)
*C. neocaninus* **T**	HMJAU 48691	China	ON254453	OR105140	Xie et al. (2025)
*C. neocaninus*	HMJAU 48690	China	ON254455	OR105141	Xie et al. (2025)
*C. nettieae* **T**	JFA9613	USA	MZ580442	—	Dima et al. (2021)
*C. ochraceodiscus* **T**	DJM2195	USA	MZ663795	—	Dima et al. (2021)
*C. parvulosquamolosus* **T**	UCH AC371	Panama	OP339753	—	Liimatainen et al. (2022b)
*C. pelerinii* **T**	XC2012-21	France	MH784627	—	Dima et al. (2021)
*C. perrotensis* **T**	TENN071126	Canada	KX897405	—	Dima et al. (2021)
*C. perrotensis*	HMJAU 48662	China	OR140843	OR140846	Xie et al. (2025)
*C. perrugatus*	771	Italy	JF907864	—	Osmundson et al. (2013)
*C. perviolaceus* **T**	FLAS-F32992	USA	MZ580438	—	Dima et al. (2021)
*C. qilianensis* **T**	HMJAU 44508	China	ON254416	OR105102	Xie et al. (2025)
*C. qilianensis*	HMJAU 44509	China	ON254417	OR105103	Xie et al. (2025)
*C. rarus* **T**	DBB04712	USA	MZ663800	—	Dima et al. (2021)
*C. rattinoides* **T**	PDD88283	New Zealand	JX000375	JX000406	Soop et al. (2018)
*C. robustianomalus* **T**	HMAS 254763	China	ON254459	OR105145	Xie et al. (2025)
*C. robustianomalus*	HMAS 254764	China	ON254460	OR105146	Xie et al. (2025)
*C. rufolilacinus* **T**	HMJAU 48739	China	ON254456	OR105142	Xie et al. (2025)
*C. rufolilacinus*	HMJAU 48745	China	ON254457	OR105143	Xie et al. (2025)
*C. sclerophyllarum* **T**	HO-A20430A6	Australia	AY669637	AY669637	Garnica et al. (2005)
*C. sericeolazulinus* **T**	JFA12053	Costa Rica	EF420146	—	Ammirati et al. (2007)
*C. subalbocyaneus*	HMJAU 44282	China	ON254424	OR105111	Xie et al. (2025)
*C. subalbocyaneus* **T**	HMJAU 48659	China	ON254425	OR105112	Xie et al. (2025)
*C. subanomalus*	HMAS 277626	China	ON254488	OR105174	Xie et al. (2025)
*C. subanomalus* **T**	HMJAU 48752	China	ON254489	OR105175	Xie et al. (2025)
*C. subclackamasensis*	HMAS 291362	China	ON254394	OR105082	Xie et al. (2025)
*C. subclackamasensis*	HMAS 281433	China	ON254395	OR105083	Xie et al. (2025)
*C. suecicolor* **T**	PDD74698	New Zealand	JX000360	JX000391	Soop et al. (2018)
*C. tabularis*	HMJAU 44248	China	ON254468	OR105154	Xie et al. (2025)
*C. tabularis* **T**	CFP949	Sweden	KX302275	—	Dima et al. (2016)
*C. tetonensis* **T**	JFA10350	USA	MZ580436	—	Dima et al. (2021)
*C. tropicus* **T**	tcqushi006	China	OQ913379	—	Xie et al. (2025)
*C. tropicus*	Li 150728-56	China	OQ913380	—	Xie et al. (2025)
*C. vernalianomalus* **T**	HMJAU 48770	China	ON254487	OR105173	Xie et al. (2025)
*C. violaceobrunneus*	UCH AC41	Panama	OP265182	—	Liimatainen et al. (2022b)
*C. wudihuensis*	KUN-HKAS 151737	China	PZ274244	PZ274247	This study
*C. wudihuensis* **T**	KUN-HKAS 151738	China	PZ274245	PZ274248	This study
*C. xizangensis*	HMAS 275210	China	ON254495	OR105177	Xie et al. (2025)
*C. xizangensis* **T**	HMAS 274227	China	ON254496	—	Xie et al. (2025)

To construct the phylogenetic tree based on the dataset of subg. *Leprocybe*, maximum likelihood (ML) analysis was conducted using RAxML-HPC2 v.8.2.12 (Miller et al. 2010) under the GTRCAT model with 1,000 rapid bootstrap replicates. For Bayesian inference (BI), the optimal evolutionary models were determined using MrModeltest v.2.3 (Nylander et al. 2004) based on the Akaike information criterion (AIC). The GTR+I+G and GTR models were selected for the ITS and nrLSU regions, respectively. BI was performed using MrBayes v.3.2 with four simultaneous Markov chains running for 1,240,000 generations, sampling every 100 generations. The first 25% of trees were discarded as burn-in. Convergence was assumed when the standard deviation of split frequencies fell below 0.01 and the effective sample size (ESS) exceeded 200. The final phylogenetic trees were visualized using FigTree v.1.4.4 (Rambaut 2018) and refined in Adobe Illustrator CC 2018 (Adobe, USA).

To construct the phylogenetic tree based on the dataset of sect. *Anomali*, ML analysis was conducted using RAxML-HPC2 v.8.2.12 (Miller et al. 2010) under the GTRCAT model with 1,000 rapid bootstrap replicates. For BI, the optimal evolutionary models were determined using MrModeltest v.2.3 (Nylander et al. 2004) based on the AIC. The HKY+I+G and GTR+I+G models were selected for the ITS and nrLSU regions, respectively. BI was performed using MrBayes v.3.2 (Ronquist et al. 2012) with four simultaneous Markov chains running for 2,610,000 generations, sampling every 100 generations. The first 25% of trees were discarded as burn-in. Convergence was assumed when the standard deviation of split frequencies fell below 0.01, and the ESS exceeded 200. The final phylogenetic tree was visualized using FigTree v.1.4.4 and refined in Adobe Illustrator CC 2018 (Adobe, USA).

## Results

### Molecular analyses

**Figure 1.**
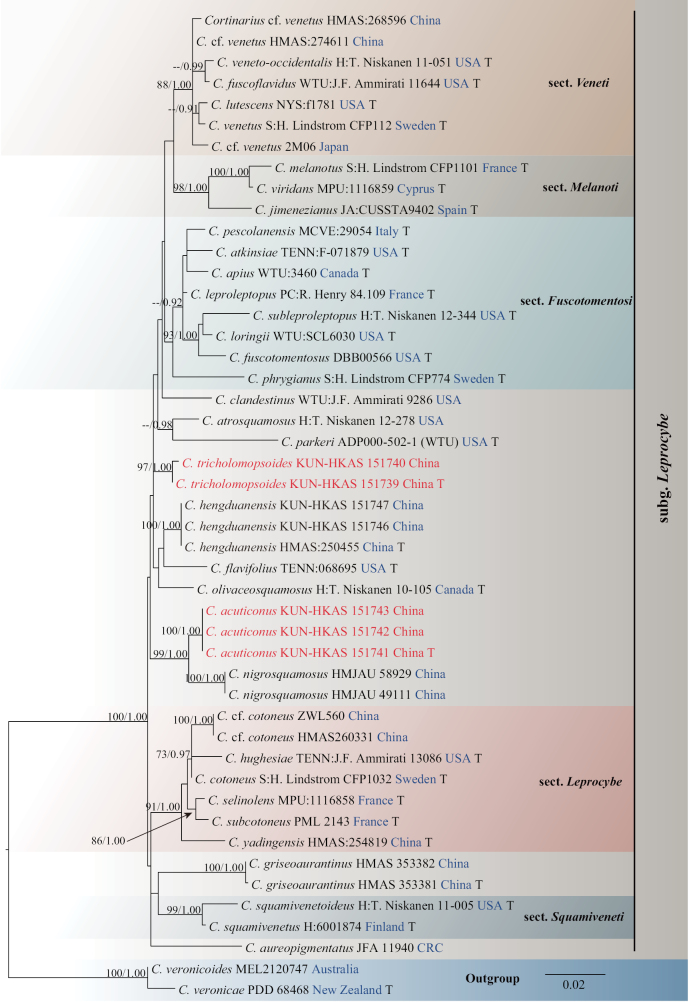
Phylogenetic tree of *Cortinarius* subg. *Leprocybe* generated from the concatenated nrITS and nrLSU dataset using maximum likelihood (ML) and Bayesian inference (BI) analyses. The dataset comprises 56 sequences representing 37 taxa, with *Cortinarius veronicoides* (MEL2120747) and *C. veronicae* (PDD 68468) designated as outgroup taxa following Hong et al. (2024). Maximum likelihood bootstrap (ML-BP) ≥ 70% and Bayesian posterior probabilities (BI-PP) ≥ 0.90 are indicated above the nodes. “-” indicates that the support value was less than the respective threshold. The specimen vouchers and country are indicated after the species names. New samples collected in this study are indicated in red. “T” indicates type specimens.

In Fig. 1 and Table 1, the concatenated dataset comprised 56 sequences (47 ITS and nine nrLSU) representing 37 taxa (47 vouchers), including 42 publicly available sequences and 14 newly generated sequences. *Cortinarius veronicoides* (MEL2120747) and *C. veronicae* (PDD 68468) were designated as outgroup taxa following Hong et al. (2024). The aligned matrix comprised 1,508 aligned nucleotide positions (ITS: 1–660; nrLSU: 661–1,508). GenBank accession numbers for all sequences are listed in Table 1. ML and BI analyses yielded congruent topologies; the ML tree was selected for phylogenetic representation (Fig. 1). Phylogenetic analyses provided robust support for the taxonomic independence of *Cortinarius tricholomopsoides* and *C. acuticonus*. Both taxa formed statistically well-supported monophyletic clades within subg. *Leprocybe*.

**Figure 2.**
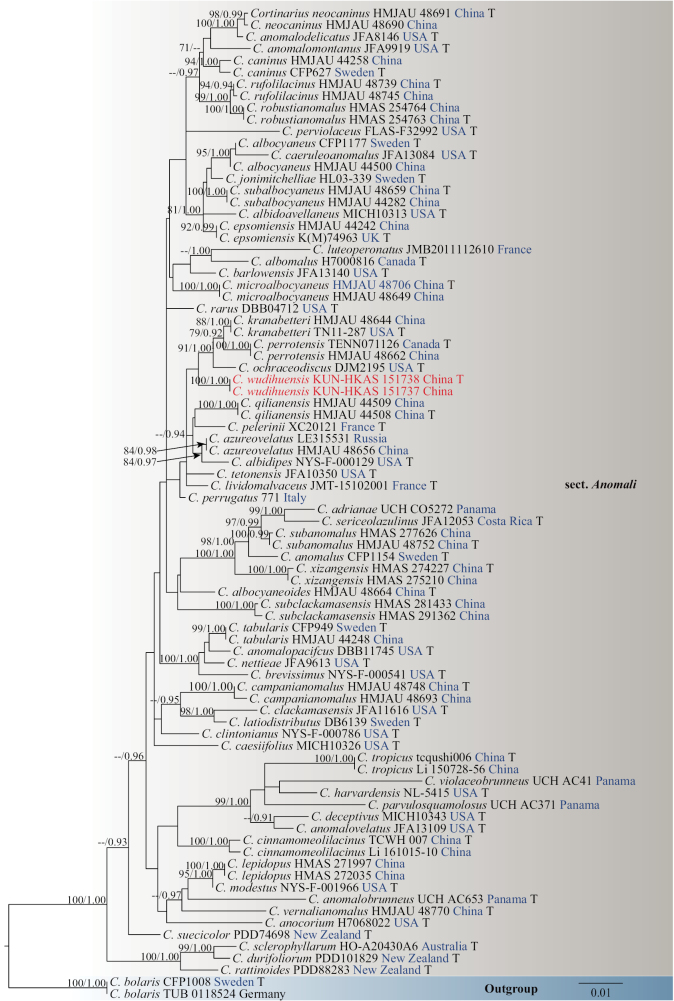
Phylogenetic tree of *Cortinarius* sect. *Anomali* generated from the concatenated nrITS and nrLSU dataset using maximum likelihood (ML) and Bayesian inference (BI) analyses. The dataset comprises 120 sequences representing 63 taxa, with *Cortinarius bolaris* (CFP1008) and *C. bolaris* (TUB 0118524) designated as outgroup taxa following Xie et al. (2025). Maximum likelihood bootstrap (ML-BP) ≥ 70% and Bayesian posterior probabilities (BI-PP) ≥ 0.90 are indicated above the nodes. “-” indicates that the support value was less than the respective threshold. The specimen vouchers and country are indicated after the species names. New samples collected in this study are indicated in red. “T” indicates type specimens.

In Fig. 2 and Table 2, the concatenated dataset comprised 120 sequences (84 ITS, 36 nrLSU) representing 63 taxa (84 vouchers), including 116 publicly available sequences and four newly generated sequences. Following the study by Xie et al. (2025), *Cortinarius bolaris* (CFP1008) and *C. bolaris* (TUB 0118524) were designated as outgroup taxa. The aligned matrix comprised 1,505 aligned nucleotide positions (ITS: 1–613; nrLSU: 614–1,505). GenBank accession numbers for all sequences are listed in Table 2. ML and BI analyses yielded congruent topologies; therefore, the ML tree was selected for phylogenetic representation (Fig. 2). Phylogenetic analyses provided robust support for the taxonomic independence of *Cortinarius wudihuensis*. This taxon formed a statistically well-supported monophyletic clade within sect. *Anomali*.

### Taxonomy

#### Cortinarius acuticonus

Taxon classificationFungiAgaricalesCortinariaceae

Y.W. Yang, Y. Zhang & Q. Zhao sp. nov.

67B4C043-4701-5BB5-A4AE-91FD1F223E2C

Index Fungorum: IF905217

Fig. 3

##### Chinese name.

细锥鳞丝膜菌 (xi zhui lin si mo jun).

##### Etymology.

“*acuticonus*” refers to the acutely conical shape of the pileus.

##### Holotype.

**China** • Yunnan Province: Dali, Yongping County, Jinguangsi Nature Reserve at 25.321450°N, 99.886342°E, alt. 2449 m, in *Castanopsis* spp. forest, 8 Jul. 2009, JGS-15-03 (KUN-HKAS 151741).

##### Diagnosis.

*Cortinarius acuticonus* differs from *C. nigrosquamosus* by its acutely conical pileus entirely lacking olive tinges, covered with much finer and more densely arranged black, Strobilomyces-like scales, a distinct membranous annulus on the stipe, and broadly ellipsoid to ellipsoid basidiospores (6–8.5 × 4.5–6.5 μm). Molecularly, it is distinguished by 13 significant base-pair differences in the sequence alignment.

**Figure 3.**
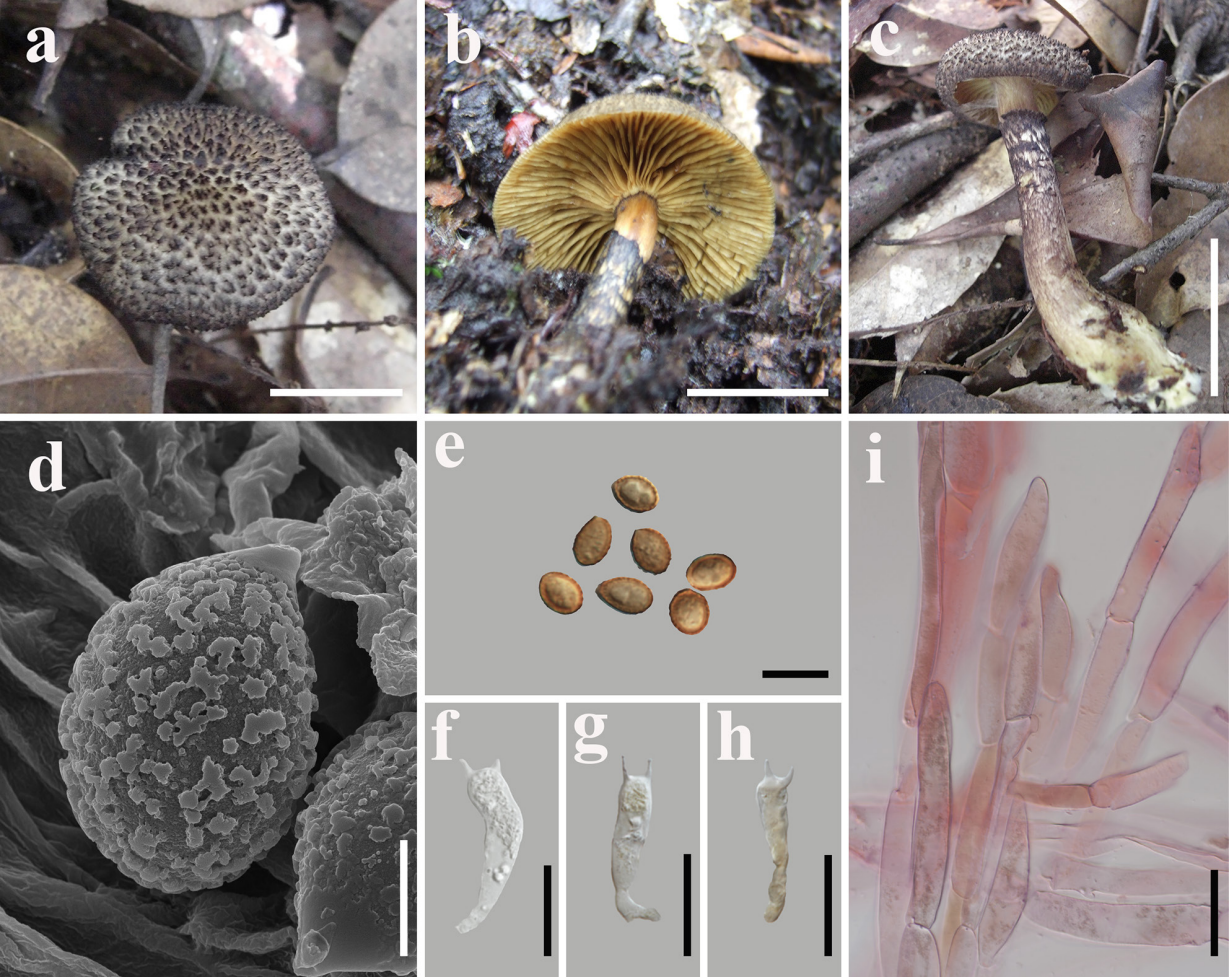
*Cortinarius acuticonus*. **a–c**. Basidiomata (**a, c**. KUN-HKAS 151741, holotype; **b**. KUN-HKAS 151742); **d**. Scanning electron micrograph of basidiospore; **e**. Basidiospores; **f–h**. Basidia; **i**. Epicutis. Scale bars: 1 cm (**a, b**); 2 cm (**c**); 2 μm (**d**); 10 μm (**e**); 20 μm (**f–i**).

##### Macrostructures.

***Basidiomata*** medium-sized. ***Pileus*** 2–4 cm diam., initially acutely conical, becoming hemispherical to applanate with age; surface reddish brown (8E4) at the disc, fading to reddish grey (7B2) towards the margin, entirely covered with black (G1) scales resembling ***Strobilomyces***. *Context* 2–6 mm thick, brown (7E5). ***Lamellae*** adnexed, 3–5 mm broad, moderately crowded, unequal, greyish yellow (4C5) to yellowish brown (5D6); edge undulating. *Stipe* 40–70 × 5–10 mm, clavate, base slightly bulbous; surface pale yellow (4A3) at the apex, brown (6C5) to brownish orange (6E4) in the middle, becoming paler downwards, covered with black (G1) fibrils, with a membranous annulus; longitudinally striate. ***Basal mycelium*** yellowish white (2A2), distinctive.

##### Microstructures.

***Basidiospores*** [90/3/3] (6–) 6.5–8 (–8.5) × (4.5–) 5–6 (–6.5) μm [Q = (1.05–) 1.15–1.47 (–1.55), **Q** = 1.3 ± 0.1], broadly ellipsoid to ellipsoid, cinnamon in 10% KOH, light orange-brown, and distinctly verrucose. ***Basidia*** 20–38 × 5.5–9 μm, clavate, thin-walled, mostly subhyaline, 4-spored, colorless or with yellowish contents. ***Lamella trama*** hyphae 4.5–6.5 μm diam., smooth, colorless. ***Lamella edges*** heterogeneous, with sterile cells, 17–23.5 × 5–7 μm, clavate, subhyaline. ***Pleurocystidia*** absent. ***Pileipellis*** duplex: epicutis well developed, hyphae interwoven, with some ascending fascicles, 5.5–14.5 μm diam., cylindrical, colorless or with greyish-black pigment; Hypodermium composed of uniform, moniliform, tightly interwoven, hyaline cells, hyphae 7–21 μm diam. ***Stipe hyphae*** 7.5–13 μm diam., colorless, smooth. ***Clamp connections*** present.

##### Habitat.

Scattered or gregarious on the ground in *Castanopsis orthacantha* forest.

##### Distribution.

Known from the western Yunnan Province, China.

##### Additional material examined.

**China** • Yunnan Province: Dali, Yongping County, Jinguangsi Nature Reserve at 25.351003°N, 99.860261°E, alt. 2490 m, in *Castanopsis orthacantha* forest, 8 Sep. 2009, JGS-27-11 (KUN-HKAS 151742); **China** • Yunnan Province: Dali, Yongping County, Jinguangsi Nature Reserve at 25.321450°N, 99.886342°E, alt. 2449 m, in *Castanopsis orthacantha* forest, 5 Aug. 2009, JGS-18-17 (KUN-HKAS 151743).

##### Notes.

*Cortinarius acuticonus* is a striking species that is easily recognized in the field, characterized by an acutely conical pileus densely covered with black, *Strobilomyces*-like scales, a distinct membranous annulus on the stipe, and broadly ellipsoid to ellipsoid basidiospores (6–8.5 × 4.5–6.5 μm). Morphologically, it can be easily confused with *C. nigrosquamosus*. The latter is another dark-scaled species previously recorded in Shangri-La Pudacuo National Park and discussed in recent comprehensive studies of the genus *Cortinarius* in China (Xie 2022). However, *C. acuticonus* can be distinguished from *C. nigrosquamosus* by the absence of an olive tinge on its pileus surface. Additionally, the scales on the pileus of *C. acuticonus* are finer and more densely arranged than those of *C. nigrosquamosus*. At the molecular level, sequence alignment between *C. acuticonus* and *C. nigrosquamosus* reveals 13 significant base-pair differences, providing strong evidence for recognizing *C. acuticonus* as a distinct species. Furthermore, the collection sites for *C. acuticonus* have an average elevation of 2460 m, which is lower than the 3500 m for *C. nigrosquamosus*. This altitudinal difference may be a contributing factor to the morphological and molecular divergences observed between the two species.

#### Cortinarius tricholomopsoides

Taxon classificationFungiAgaricalesCortinariaceae

Y.W. Yang, Y. Zhang & Q. Zhao sp. nov.

5049F896-E786-5EC7-AE3F-A8CD4FFBB148

Index Fungorum: IF905218

Fig. 4

##### Chinese name.

拟口蘑丝膜菌 (ni kou mo si mo jun).

##### Etymology.

“*tricholomopsoides*” refers to the resemblance of this new species to the genus *Tricholomopsoides*.

##### Holotype.

**China** • Yunnan Province: Lijiang City, Laojunshan Nature Reserve, at 26.631426°N, 99.719085°E, alt. 3960 m, in *Abies* forest, 26 Jul. 2018, LJ1334 (KUN-HKAS 151739).

##### Diagnosis.

*Cortinarius tricholomopsoides* is macromorphologically reminiscent of the European species *C. cotoneus* due to its tomentose to squamulose pileal surface but can be distinctively separated by its pale yellow to light brown pileal coloration entirely lacking olivaceous pigments throughout the basidiocarp, the presence of greyish-orange squamules on the stipe surface, and its negligible odor, slightly smaller, narrower basidiospores (7–9 × 6–7.5 µm).

##### Macrostructures.

***Basidioma*** large-sized. ***Pileus*** 5–10 cm diam., initially hemispherical, becoming applanate; surface initially pastel yellow (3A4), becoming light brown (7D5), covered with light-brown (7D5) squamules, dense at the disc, sparse towards the margin; context 1–4 mm thick, yellowish white (1A2). ***Lamellae adnexed***, 5–7 mm broad, moderately crowded, unequal, greyish yellow (1B3); edge entire. ***Stipe*** 80–120 × 8–20 mm, cylindrical, tapering upwards; surface greyish yellow (2B3), covered with greyish orange (5B6) fibrillose squamules, with a cortinate annular zone, longitudinally striate. ***Basal mycelium*** white (A1). ***Odor*** not distinctive.

**Figure 4.**
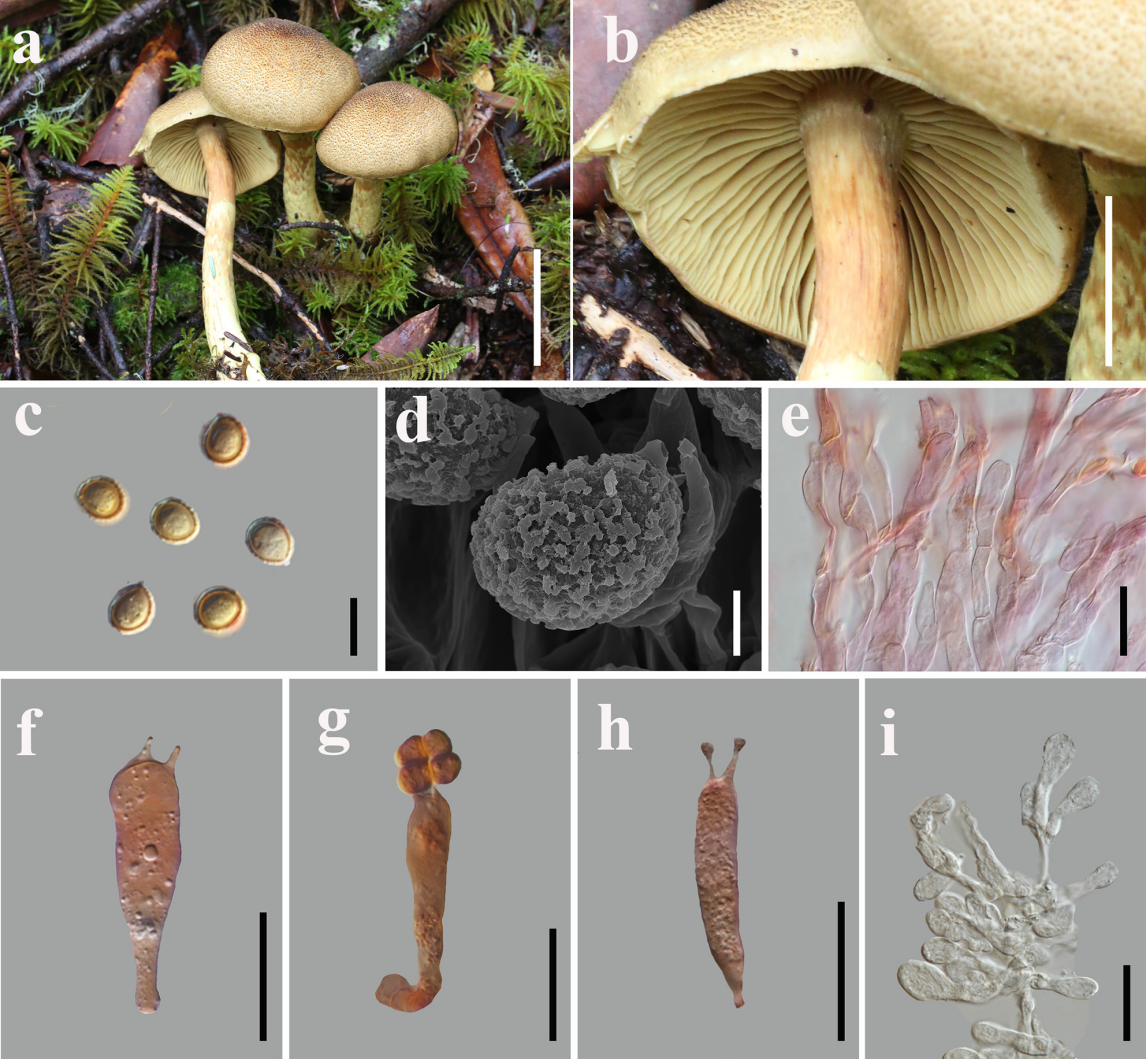
*Cortinarius tricholomopsoides*. **a, b**. Basidiomata (**a**. KUN-HKAS 151739, holotype; **b**. KUN-HKAS 151740); **c**. Basidiospores; **d**. Scanning electron micrograph of basidiospore; **e**. Epicutis; **f–h**. Basidia; **i**. Lamella edges. Scale bars: 5 cm (**a**); 2 cm (**b**); 10 μm (**c**); 2 μm (**d**); 20 μm (**e–i**).

##### Microstructures.

***Basidiospores*** [60/2/2] (7–) 7.5–9 (–9.5) × (6–) 6.5–7.5 (–8) μm [Q = (1.04–) 1.08–1.4 (–1.51), Q = 1.24 ± 0.09], subglobose to ellipsoid, cinnamon in 10% KOH, light brownish yellow, and distinctly verrucose. ***Basidia*** 26.5–46.5 × 5.5–10.5 μm, clavate, thin-walled, mostly subhyaline, 4-spored. ***Lamella edges*** heterogeneous, with sterile cells, 16–26 × 5.5–8.5 μm, clavate, subhyaline, thin-walled. ***Lamella trama***, colorless or with yellowish contents, hyphae 4.5–6.5 μm diam. ***Pleurocystidia*** absent. ***Pileipellis*** duplex: epicutis well developed, hyphae interwoven, with some ascending fascicles, 4.5–12 μm diam., cylindrical, colorless or with yellowish contents; hypocutis composed of cylindrical, strongly interwoven, colorless or with yellowish contents, hyphae 5.5–9 μm diam. *Stipe hyphae* 8.5–12.5 μm diam., colorless, smooth. ***Clamp connections*** present.

##### Habitat.

Scattered or gregarious on the ground in the *Abies* forest.

##### Distribution.

Known from the Northwestern Yunnan Province, China.

##### Additional material examined.

**China** • Yunnan Province: Lijiang City, Laojunshan Nature Reserve, at 26.631426°N, 99.719085°E, alt. 3960 m, in *Abies* forest, 27 Aug. 2018 LJ1727 (KUN-HKAS 151740).

##### Notes.

*Cortinarius tricholomopsoides* is morphologically reminiscent of *C. cotoneus*, both species characterized by tomentose to squamulose pileal surfaces. However, *C. cotoneus* displays characteristic olivaceous to brownish-olivaceous pigmentation throughout the basidiocarp and produces a pronounced raphanoid odor (Bidaud et al. 2021). Conversely, *C. tricholomopsoides* has pale yellow to light brown pileal coloration with grayish-orange squamulation on the stipe surface while exhibiting negligible olfactory characteristics. Additionally, the stipe of *C. cotoneus* is typically clavate to bulbous, whereas *C. tricholomopsoides* has a cylindrical stipe that tapers upward. Microscopically, *C. cotoneus* generally possesses slightly larger and broader basidiospores (typically 8–9.5 × 7–8 µm) compared to those of *C. tricholomopsoides* (7–9 × 6–7.5 µm).

#### Cortinarius wudihuensis

Taxon classificationFungiAgaricalesCortinariaceae

Y.W. Yang, Y. Zhang & Q. Zhao sp. nov.

4916A536-EEB4-5FF3-A735-1044BE466639

Index Fungorum: IF905440

Fig. 5

##### Chinese name.

无底湖丝膜菌 (wu di hu si mo jun).

##### Etymology.

“*wudihuensis*” refers to the type locality, Wudi Lake, a famous lake in Shangri-La City, China.

##### Holotype.

**China** • Yunnan Province: Shangri-La City, Potatso National Park, Wudi Lake at 28.249444°N, 99.977709°E, alt. 3850.3 m, in *Quercus aquifolioides* forest, 8 Aug. 2025, Zyue-121 (KUN-HKAS 151738).

##### Diagnosis.

*Cortinarius wudihuensis* is macromorphologically most similar to *C. microalbocyaneus*, described from Northeast China, due to its small- to medium-sized basidiomata and a shared color transition from violaceous to brownish tones. However, *C. wudihuensis* can be distinctively distinguished by its slightly larger basidiospores (7–9 × 5.5–7 μm), a brown pileus covered with white fibrils that tends to crack with age, and a stipe featuring a distinct brown ring-like zone.

**Figure 5.**
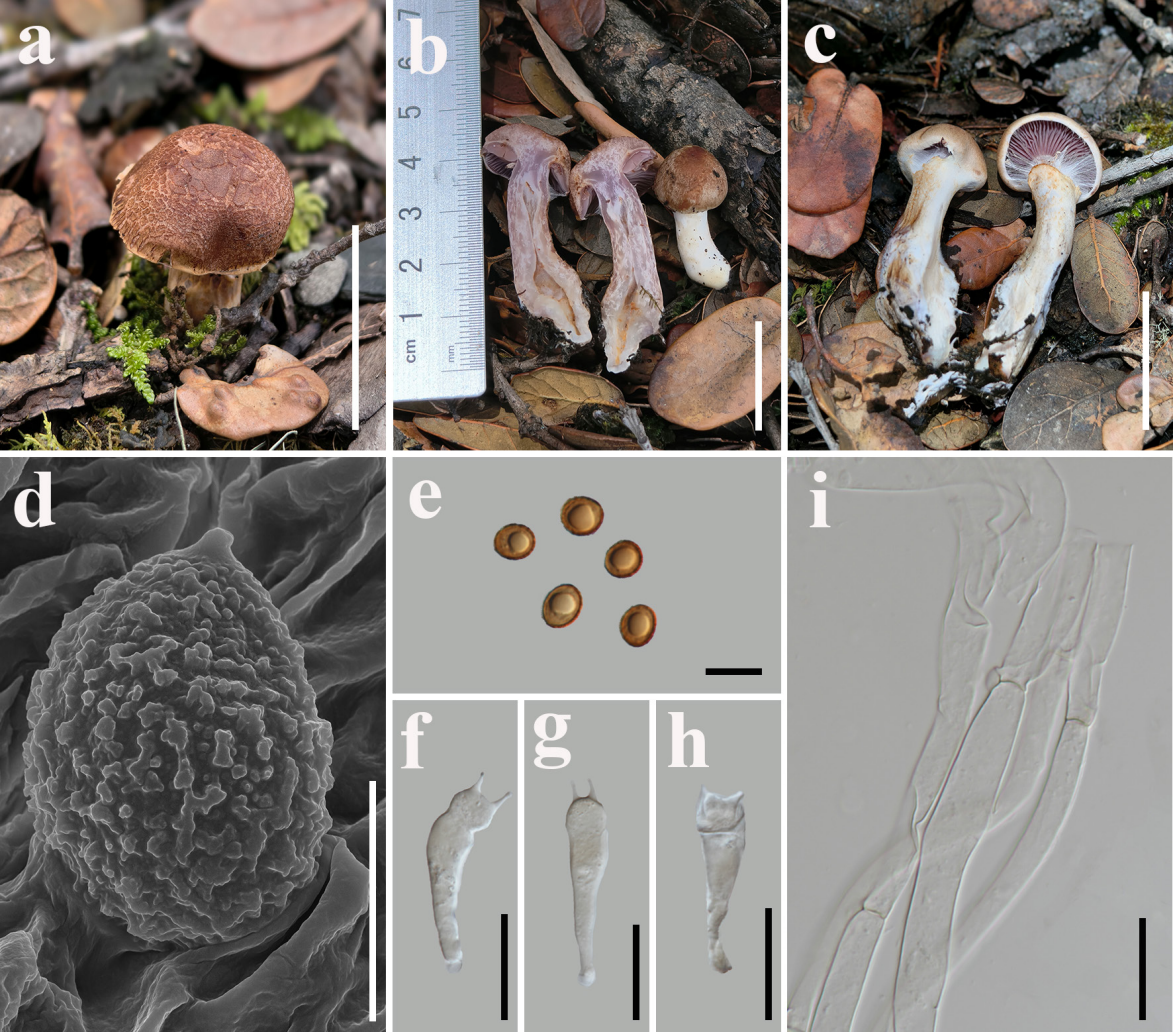
*Cortinarius wudihuensis*. **a, b**. Basidiomata (**a, b**. KUN-HKAS 151738, holotype; **c**. KUN-HKAS 151737); **d**. Scanning electron micrograph of basidiospore; **e**. Basidiospores; **f–h**. Basidia; **i**. Universal veil hyphae. Scale bars: 2 cm (**a–c**); 5 μm (**d**); 10 μm (**e**); 20 μm (**f–i**).

##### Macrostructures.

***Basidioma*** small- to medium-sized. ***Pileus*** 1–3 cm diam., initially hemispherical, becoming applanate; surface initially brown (7D7) covered with white (A1) fibrillose, becoming light brown (7D6), covered with light brown (7D6) squamules, surface prone to cracking; context 1–6 mm thick, marbled with dull violet (15D4) and pale violet (15A3). ***Lamellae adnexed***, 2–4 mm broad, moderately crowded, unequal, dull violet (15D4) to pasted violet (15A4); edge entire. *Stipe* 20–50 × 7–12 mm, slightly thickened at the base, surface white, upper part with greyish violet (15E6) tinges, a brown (7C4) ring-like zone at the middle to upper part, internal part somewhat hollow. ***Basal mycelium*** white (A1). ***Universal veil*** white. ***Odor*** not distinctive.

##### Microstructures.

***Basidiospores*** [60/2/2] (6–) 7–9 × (5–) 5.5–7 (–7.5) μm [Q = (1.01–) 1.06–1.37 (–1.43), **Q** = 1.21 ± 0.1], subglobose to ellipsoid, cinnamon in 10% KOH, reddish brown, and distinctly verrucose. ***Basidia*** 28.5–44 × 6.5–12 μm, clavate, thin-walled, mostly subhyaline, 4-spored. ***Lamella edges*** heterogeneous, with sterile cells, 21–29 × 7–8 μm, densely arranged, clavate, subhyaline, thin-walled. ***Lamella trama***, colorless, hyphae 3–4.5 μm diam. ***Pleurocystidia*** absent. ***Pileipellis*** duplex: epicutis thinly to moderately developed, hyphae interwoven, yellowish to yellowish brown hyphae, 3.5–5.5 μm wide, smooth to slightly encrusted; hypocutis well developed, hyphae 5–7 μm wide, hyaline or slightly yellowish brown. ***Stipe hyphae*** 4–5.5 μm diam., colorless, smooth. ***Veil hyphae***, 6–9.5 μm diam. ***Clamp connections*** present.

##### Habitat.

Scattered or gregarious on the ground in the *Quercus aquifolioides* forest.

##### Distribution.

Known from the Northwestern Yunnan Province, China.

##### Additional material examined.

**China** • Yunnan Province: Shangri-La City, Potatso National Park, Wudi Lake at 28.249436°N, 99.977669°E, alt. 3867.4 m, in *Quercus aquifolioides* forest, 8 Aug. 2025, XDY-107 (KUN-HKAS 151737).

##### Notes.

*Cortinarius wudihuensis* is characterized by its small to medium-sized basidiomata, a brown pileus covered with white fibrils that tends to crack with age, and a stipe featuring a distinct brown ring-like zone. Morphologically, it is most similar to *C. microalbocyaneus*, described from Northeast China (Xie et al. 2025). Both species share small basidiospores and a similar color transition from violaceous to brownish tones. However, *C. wudihuensis* can be distinguished by its slightly larger basidiospores (7–9 × 5.5–7 μm vs. 6.8–7.2 × 5.8–6.2 μm in *C. microalbocyaneus*) and its specific habitat in Shangri-La, potentially associated with *Quercus aquifolioides*. Phylogenetically, it belongs to sect. *Anomali*, where many small-sized species show high morphological convergence.

## Discussion

The morphological characteristics of *Cortinarius* subg. *Leprocybe* have historically been regarded as relatively stable and distinct (Fries 1838). In classical morphological taxonomy, species delimitation within subg. *Leprocybe* has primarily depended on a suite of diagnostic characters, including dry, non-hygrophanous pilei with tomentose to squamulose surfaces, subglobose to broadly ellipsoid verrucose basidiospores, and the presence of fluorescent pigments observable under ultraviolet light (Ammirati et al. 2021; Bidaud et al. 2021). However, reliance on isolated characters is frequently inadequate for delineating discrete species, as these traits may demonstrate intraspecific variation or represent convergent evolution among phylogenetically distant lineages. For instance, *C. venetus* from Europe, *C. veneto-occidentalis* from North America, and *C. hengduanensis* from China all share similar olive tones despite their geographic separation (Ammirati et al. 2021; Bidaud et al. 2021). The absence of olive tones in *C. tricholomopsoides* is particularly noteworthy (Ammirati et al. 2021). Although green or olive fluorescence attributed to leprocybin compounds has been traditionally regarded as a diagnostic characteristic for subgenus classification, the identification of *C. acuticonus* contributes to accumulating phylogenetic evidence indicating that olive phenotypic expression is not ubiquitous within this taxonomic subgenus. Recent taxonomic revisions in North America and Europe have established significant continental endemism within subg. *Leprocybe* (Bidaud et al. 2021). The present phylogenetic analyses corroborate this biogeographical framework, demonstrating that the examined taxa represent phylogenetically divergent lineages relative to their European and American congeners. Ecologically, the newly described species exhibit specialized mycorrhizal partnerships: *C. tricholomopsoides* demonstrates an obligate association with *Abies* spp., while *C. acuticonus* maintains exclusive symbiotic relationships with *Castanopsis* spp. These host-fungus associations differ substantially from the ecological requirements documented for European representatives of subg. *Leprocybe*, which typically form mycorrhizal associations with *Fagus* or *Quercus* in temperate deciduous forests (Ammirati et al. 2021; Bidaud et al. 2021).

Similar severe taxonomic challenges are also evident in *Cortinarius* sect. *Anomali*. Although this section is species-rich and widely distributed, most known species were originally described from Europe and North America, and its species diversity in Asia has long been severely underestimated (Xie et al. 2025). The introduction of molecular phylogenetics has revealed extremely high cryptic diversity within this section and corrected many historical morphological misidentifications; for example, the widely reported *C. anomalus* has not actually been found in China, with many specimens turning out to be *C. subclackamasensis* or other species (Dima et al. 2016, 2021; Xie et al. 2025). Correctly identifying species in sect. *Anomali* faces significant challenges, primarily because macro- and micromorphological characteristics overlap extensively across species (Dima et al. 2016, 2021; Xie et al. 2025). Species in this section generally possess violaceous to blue tinges in early stages, which often fade to grayish brown or brown upon maturity (Dima et al. 2021). Furthermore, unlike European species that typically have a whitish universal veil, the veil color of Chinese species is highly variable (Dima et al. 2016, 2021). Within this complex taxonomic context, basidioma size and microscopic features serve as key clues for differentiating *Anomali* species. *Cortinarius wudihuensis*, newly described in this study, belongs to this section. Although it closely resembles *C. microalbocyaneus* in its small basidiomata and the color transition from violaceous to brownish tones, molecular evidence indicates that *C. wudihuensis* constitutes a completely independent phylogenetic lineage (Xie et al. 2025).

The identification of *C. acuticonus*, *C. tricholomopsoides*, and *C. wudihuensis* in subalpine forests of northwestern Yunnan highlights the severely underestimated diversity of *Cortinarius* in the province’s alpine regions. In recent years, 36 new species of *Cortinarius* have been discovered in this region, including two species belonging to subg. *Leprocybe* and 11 species belonging to sect. *Anomali* (Zhang et al. 2023; Zhou et al. 2023; Hong et al. 2024; Jia et al. 2025; Wang et al. 2025; Xie et al. 2025; Yang et al. 2025; Li et al. 2026; Zhou et al. 2026). It is worth noting that while the concatenated dataset yielded highly resolved and statistically robust topologies for the new taxa, a limitation of this study lies in the incomplete coverage of nrLSU sequences for several reference species retrieved from public databases. Due to the historical reliance on ITS as the primary fungal barcode (Horak 1987; Shao and Xiang 1997; Liimatainen 2014; Liimatainen et al. 2015, 2017, 2020, 2022a), many earlier collections and type descriptions lack complementary nrLSU data, resulting in missing data zones within the multi-gene matrix. Although maximum likelihood and Bayesian inference frameworks are mathematically resilient to such partial missing data, future systematic revisions would significantly benefit from expanding the sequencing coverage of protein-coding loci and complete ribosomal arrays across all historical type materials to further stabilize the deeper evolutionary branches within subg. *Leprocybe* and sect. *Anomali*. In contrast to the comprehensive taxonomic documentation of subg. *Leprocybe* in North America and Europe, only six species have previously been reported from China, namely *C. cotoneus*, *C. hengduanensis*, *C. griseoaurantinus*, *C. nigrosquamosus*, *C. yadingensis*, and *C. venetus* (Keissler and Hohwag 1937; Horak 1987; Shao and Xiang 1997; Xie 2022; Hong et al. 2024; Dang et al. 2025). The taxonomic descriptions of *C. tricholomopsoides* and *C. acuticonus*, in conjunction with the recently described *C. hengduanensis*, *C. griseoaurantinus*, and *C. yadingensis*, substantially contribute to addressing the considerable knowledge deficiency concerning Asian representatives of this subgenus (Keissler and Hohwag 1937; Horak 1987; Shao and Xiang 1997; Hong et al. 2024; Dang et al. 2025). In China, 26 species of sect. *Anomali* have been previously reported. The current discovery of *C. wudihuensis* confirms that the species diversity of this section remains incompletely understood (Xie et al. 2025).

## Supplementary Material

XML Treatment for Cortinarius acuticonus

XML Treatment for Cortinarius tricholomopsoides

XML Treatment for Cortinarius wudihuensis

## Additional information

### Artificial Intelligence (AI) use

The authors accept full responsibility for the content of the manuscript, including the disclosure of any use of AI.

No AI tools were used in the preparation of this manuscript.

### Author ORCIDs

Y-W. Yang https://orcid.org/0000-0002-0257-904X

Q. Zhao https://orcid.org/0000-0001-8169-0573

Y. Zhang https://orcid.org/0009-0000-5248-0171

### Data availability

All of the data that support the findings of this study are available in the main text.
